# Integrative genomic analyses identify susceptibility genes underlying COVID-19 hospitalization

**DOI:** 10.1038/s41467-021-24824-z

**Published:** 2021-07-27

**Authors:** Gita A. Pathak, Kritika Singh, Tyne W. Miller-Fleming, Frank R. Wendt, Nava Ehsan, Kangcheng Hou, Ruth Johnson, Zeyun Lu, Shyamalika Gopalan, Loic Yengo, Pejman Mohammadi, Bogdan Pasaniuc, Renato Polimanti, Lea K. Davis, Nicholas Mancuso

**Affiliations:** 1 Yale School of Medicine, Department of Psychiatry, Division of Human Genetics, New Haven, CT USA; 2 Veteran Affairs Connecticut Healthcare System, West Haven, CT USA; 3 Division of Genetic Medicine, Department of Medicine, Vanderbilt University Medical Center, Nashville, TN USA; 4 Vanderbilt Genetics Institute, Vanderbilt University Medical Center, Nashville, TN USA; 5 Department of Integrative Structural and Computational Biology, The Scripps Research Institute, La Jolla, CA USA; 6 Bioinformatics Interdepartmental Program, University of California Los Angeles, Los Angeles, CA USA; 7 Department of Computer Science, University of California Los Angeles, Los Angeles, CA USA; 8 Department of Population and Public Health Sciences, Keck School of Medicine, University of Southern California, Los Angeles, CA USA; 9 Institute for Molecular Bioscience, The University of Queensland, Brisbane, QLD Australia; 10 Scripps Translational Science Institute, The Scripps Research Institute, La Jolla, CA USA; 11 Departments of Computational Medicine, Human Genetics, Pathology and Laboratory Medicine, David Geffen School of Medicine, University of California Los Angeles, Los Angeles, CA USA; 12 Center for Genetic Epidemiology, Keck School of Medicine, University of Southern California, Los Angeles, CA USA

**Keywords:** Genetics, Genetic association study, Viral infection

## Abstract

Despite rapid progress in characterizing the role of host genetics in SARS-Cov-2 infection, there is limited understanding of genes and pathways that contribute to COVID-19. Here, we integrate a genome-wide association study of COVID-19 hospitalization (7,885 cases and 961,804 controls from COVID-19 Host Genetics Initiative) with mRNA expression, splicing, and protein levels (n = 18,502). We identify 27 genes related to inflammation and coagulation pathways whose genetically predicted expression was associated with COVID-19 hospitalization. We functionally characterize the 27 genes using phenome- and laboratory-wide association scans in Vanderbilt Biobank (n = 85,460) and identified coagulation-related clinical symptoms, immunologic, and blood-cell-related biomarkers. We replicate these findings across trans-ethnic studies and observed consistent effects in individuals of diverse ancestral backgrounds in Vanderbilt Biobank, pan-UK Biobank, and Biobank Japan. Our study highlights and reconfirms putative causal genes impacting COVID-19 severity and symptomology through the host inflammatory response.

## Abstract (web summary)

Genome-wide association studies of COVID-19 have identified genetic loci affecting disease severity, but the mechanisms remain to be fully described. Here, the authors use genetically predicted transcriptome, splicing and proteome data to identify potential genes and pathways underlying COVID- 19 severity.

## Introduction

Coronavirus disease 2019 (COVID-19), caused by the severe acute respiratory syndrome coronavirus 2 (SARS-CoV-2), was first reported in December 2019 and rapidly progressed into a global pandemic^1^. Approximately 10–20% of patients known to be infected with the respiratory virus SARS-CoV-2 need hospitalization^2^, and among them, a fraction face significant morbidity and mortality^3^. In addition to social determinants of health, the host’s genetic background is likely to contribute in explaining such diverse clinical outcomes. While previous efforts have demonstrated the role of *ACE2* and *TMPRSS2* in host defense against COVID-19^4^, there remains limited understanding for the role of host genetics contributing to severe COVID-19 outcome variability.

Genome-wide association studies (GWAS) have provided an opportunity to characterize the role of host genetics underlying COVID-19 risk and severity^5–9^. Initial efforts^8^ identified variants associated with COVID-19 related respiratory failure at the 3p21.31 and 9q34.2 regions, providing evidence for immune- and blood-group-related mechanisms. Subsequent studies leveraging much larger preexisting genetic cohorts have since replicated these results and identified more than 15 genomic regions associated with severe COVID-19 outcomes^5–7,9^. Due to extensive linkage-disequilibrium patterns, the association signals at identified risk regions typically span multiple genes (e.g., *SLC6A20*, *LZTFL1*, *CCR9*, *FYCO1*, *CXCR6*, and *XCR1* at 3p21.31), which makes identifying target genes challenging. To this end, previous work^9^ integrated lung and whole-blood transcriptomic data together with GWAS to identify genes associated with COVID-19 severity, however, this study had limited statistical power due to the relatively small sample size (2244 critically ill patients).

**Fig. 1:**
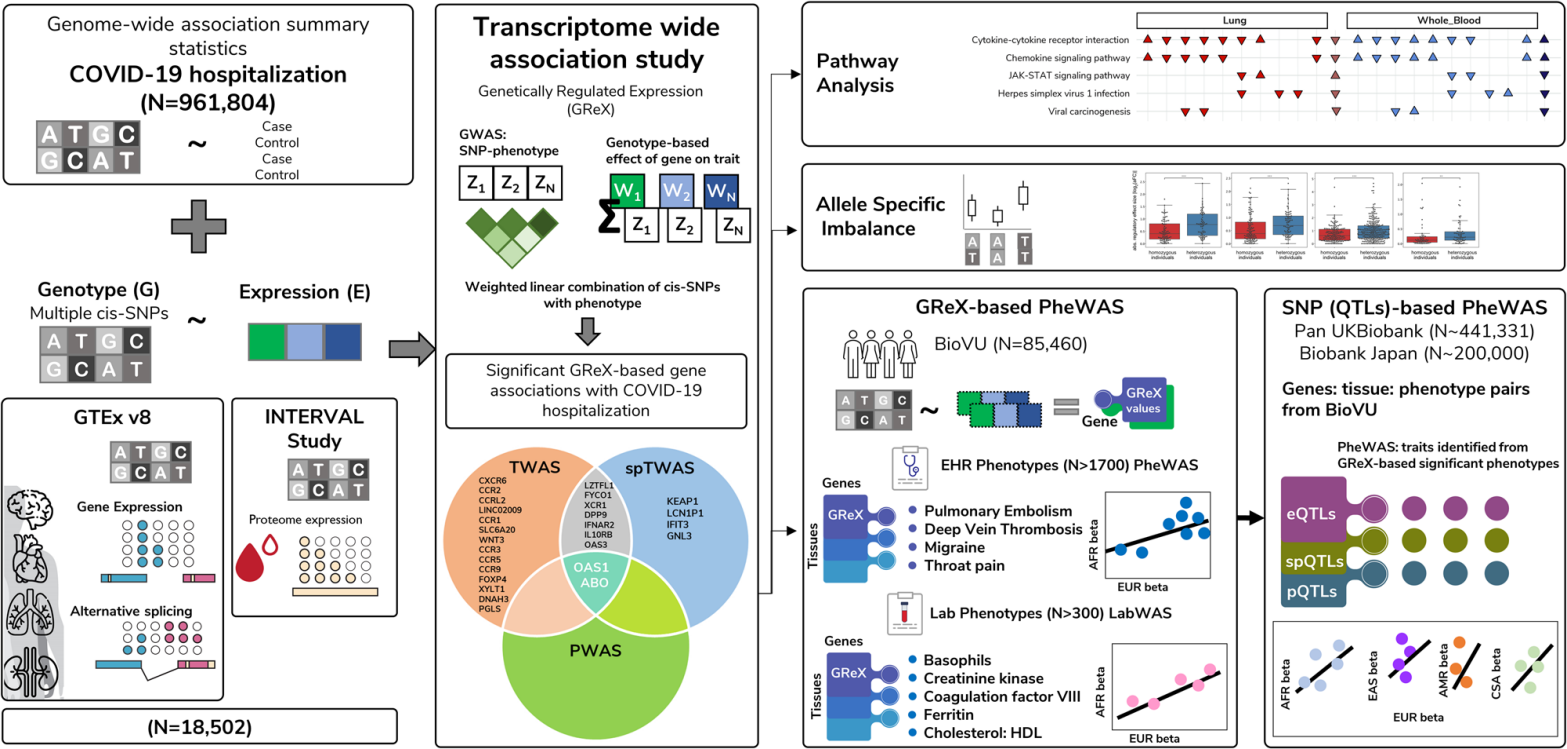
Study Overview. We performed a multilevel transcriptome-wide association study (TWAS) of genetically regulated expression (GReX) by integrating gene, splicing, and proteome expression data with genome-wide summary statistics of COVID-19 hospitalization. For the significant genes identified, we performed pathway analysis, allele-specific imbalance, and gene-based PheWAS of clinical phenotypes and LabWAS of clinical laboratory measures using individual-level GReX values in Vanderbilt Biobank (BioVU). For the significant traits identified, we performed a second, SNP-based PheWAS in multi-ancestry Pan-UKBiobank and Biobank Japan.

Here, to map genes and pathways involved in COVID-19 severity we integrate mRNA expression, splicing, and protein abundance data (*n* = 18,502) with data from a GWAS of COVID-19 related hospitalization (*n* = 7885 cases, 961,804 controls; Freeze 4 COVID-19 HGI excluding 23andMe participants^5–9^). We perform mRNA/splicing/protein transcriptome-wide association studies (TWAS/spTWAS/PWAS) to identify 27 genes across 13 genomic regions whose genetically predicted activity is associated with COVID-19 related hospitalization. We further investigate the clinical and functional role of these 27 genes using phenome-wide (PheWAS) and laboratory-wide (LabWAS) association scans to map their role in immunity and blood biomarkers in European and African ancestry patients from the Vanderbilt University Medical Center biobank (BioVU; *n* = 85,460). We replicate phenotypes identified from BioVU in secondary cohorts of multiethnic individuals from the Pan-UK Biobank (980 Admixed American, 6636 African, 8876 Central/South Asian, 2709 East Asian, 420,531 European, and 1599 Middle Eastern) and Biobank Japan (up to 200,000 Japanese participants). Taken together, our results suggest multiple molecular mechanisms contributing to severe COVID-19 outcomes and highlight potential therapeutic targets (Fig. 1).

## Results

### TWAS identifies genes for COVID-19 related hospitalization

**Fig. 2:**
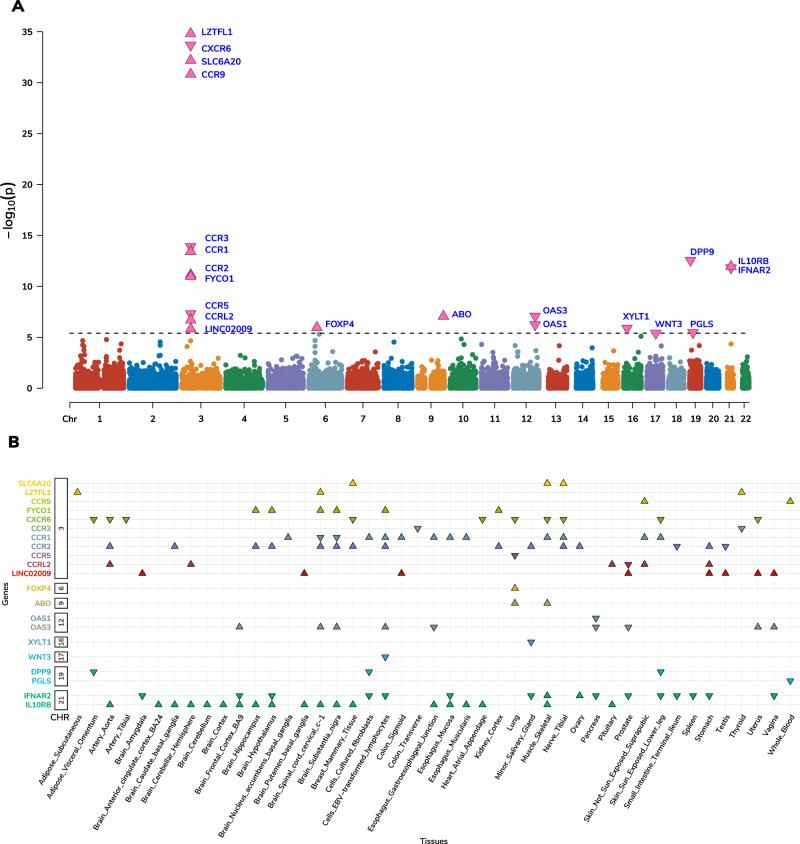
TWAS. **A** Manhattan plot of genes associated via multiple-tissue TWAS. Each data point represents a gene grouped by chromosome (x-axis) and lowest *p* value (y-axis) of the gene across significant tissues. **B** Distribution of *z*-scores across significant gene-tissue pairs. Genes are grouped based on chromosomes (y-axis) and respective tissues (x-axis). Significant genes are shown as pink triangles, wherein triangles facing up and down represent positive and negative *z*-scores, respectively.

To identify genes underlying COVID-19 related hospitalization, we tested the predicted expression of 22,207 genes across 49 tissues for association with COVID-19 related hospitalization (see Methods). We identified 123 associations representing 21 genes across 45 tissues at eight independent genomic regions (*p* value <2.3E-6; Fig. 2 and Supplementary Data 1, 2). Next, to improve statistical power, we tested for association between predicted gene expression levels from multiple tissues simultaneously with COVID-19 related hospitalization GWAS. Of the 22,207 tested genes, we identified 14 genes across ten genomic regions, which consisted of two additional genes—*XCR1* and *DNAH3* (*p* value <1.4E-06; Supplementary Fig. 1 and Supplementary Data 3). Overall, we found 23 TWAS-based gene associations across ten genomic regions.

**Fig. 3:**
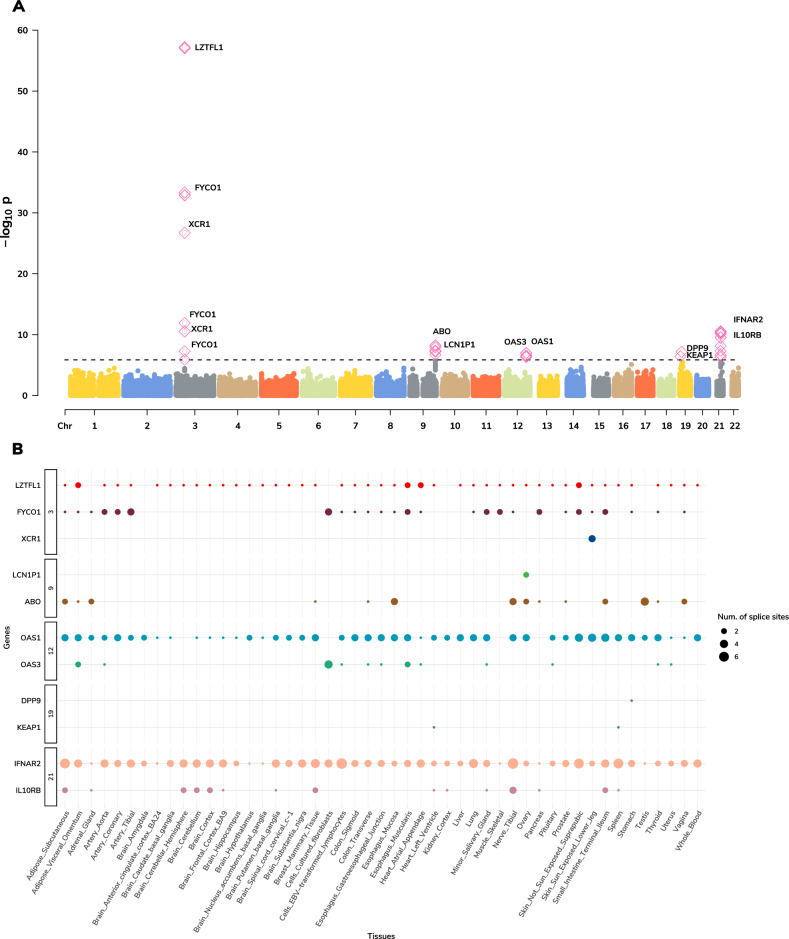
Splicing TWAS. **A** Manhattan plot of genes associated via multiple tissue spTWAS. Each data point represents a splice site grouped by chromosome (x-axis) and lowest *p* value (y-axis) of the splice site for each gene across significant tissues. The annotated genes to the splice site are labeled. Significant splice sites are shown as pink diamonds. **B** Distribution of splice sites across significant site-tissue pairs. The genes annotated to splice sites are grouped based on chromosomes (y-axis) and respective tissues (x-axis).

To find additional support for genetic regulation of identified susceptibility genes by SNPs at risk regions, we tested the 123 gene/tissue pairs identified in single tissue scans for allelic imbalance within 1 Mb GWAS regions (see Methods). We identified nine genes (*ABO*, *CCR2*, *CXCR6*, *FYCO1*, *IFNAR2*, *IL10RB*, *LZTFL1*, *OAS1*, and *OAS3*) with evidence of allelic imbalance at COVID-19 GWAS risk variants, with three genes (*ABO*, *OAS3*, and *IL10RB*; see Supplementary Fig. 2 and Supplementary Data 4) when restricted to leading GWAS index variants (*p* value <0.05/21). Together, these results further support a model where risk is conferred through transcriptomic dysregulation of key target genes. Next, we focused on the impact of alternative splicing regulation for COVID-19 severity and performed a multi-tissue splicing transcriptome-wide association study (spTWAS; see Methods). Overall, we tested 131,376 splice sites of predicted alternative-splicing expression across 49 tissues for association with COVID19 related hospitalization and identified 420 associations representing 43 splice variants for 11 genes across 49 tissues and five genomic regions (see Fig. 3 and Supplementary Data 5, 6). Next, we performed a multi-tissue analysis (see Methods) and identified 34 splice variants for 12 genes (two genes—*IFIT3* and *GNL3* not identified in single-tissue scans) across 40 tissues (*p* value <3.7E-07; see Supplementary Fig. 3 and Supplementary Data 7).

Comparing genes identified from TWAS (23 genes) and spTWAS (13 genes), nine genes were implicated by both approaches—*LZTFL1*, *DPP9*, *IL10RB*, *IFNAR2*, *OAS3*, *FYCO1*, *ABO*, *OAS1*, and *XCR1*. Alternative splicing had stronger overall association signals at the nine genes in common (*p* value = 2.17E-09), with 5/9 genes showing greater signals on average (*p* value <0.05/9; see Supplementary Data 8).

Next, we interrogated the role of genetic regulation of protein abundances and performed a proteome-wide association study (PWAS) using 1031 predictive models of plasma proteins fitted from population data in the INTERVAL study (*N* = 3301; see Methods)^10^. Of the 1031 tests performed, two genes (*ABO* and *OAS1*) were significantly associated with COVID-19 related hospitalization (*p* value <4.85E-5; Fig. 1 and Supplementary Data 9).

We employed gene set enrichment analysis (GSEA)^11^ to identify statistically overrepresented pathways for the 27 genes identified through multilevel transcriptomic integration. Significant pathways, among others, include cytokine–cytokine receptor interaction (*p*_FDR_ = 3.13E-10; Supplementary Fig. 4), chemokine signaling pathway (*p*_FDR_ = 9.89E-9), and JAK-STAT signaling pathway (*p*_FDR_ = 1.87E-2) (Supplementary Table 1). Analyzing lung and whole-blood tissue-specific TWAS *z*-scores of genes that belong to the identified pathway set, we found consistent signals of downregulation, suggesting that decreased expression levels at these genes increase the severity of COVID19 outcomes (Supplementary Fig. 4). We also performed gene regulatory network (GRN) identification, connecting transcription factors (TFs) to gene targets. Retaining TFs with most of the number of gene targets, we observed 30 TFs that target 17 genes (Supplementary Fig. 5).

Studies have reported decreased lung function in COVID-19 affected individuals. Therefore, we investigated lung traits that share a locus (colocalize) with the novel genes identified in this study, and observed *GNL3* colocalized with measures of lung function i.e., forced expiratory volume (FEV; see Methods: H_4_ PP: 93%) and forced vital capacity (FVC; H_4_ PP:89.4% Supplementary Data 10).

### Phenome- and laboratory-wide association scans highlight functional role for the 27 genes

**Fig. 4:**
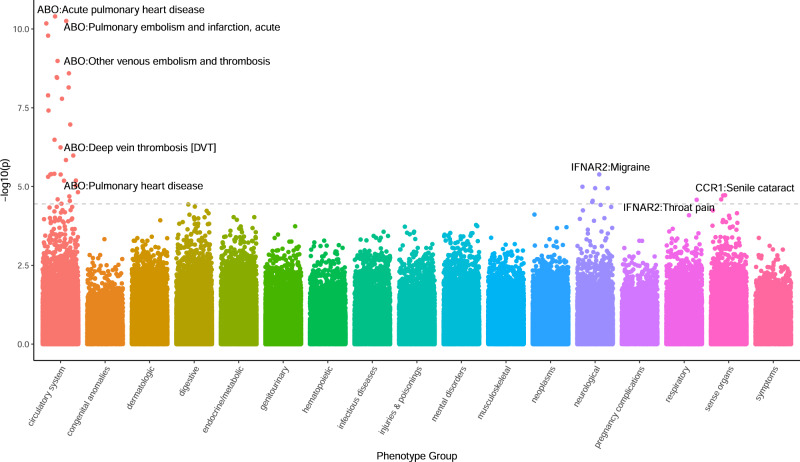
PheWAS Manhattan Plot. Each data point represents phenotypic associations with the genetically regulated expression of gene-tissue pairs. The data points are grouped and color-coded by phenotype groups (x-axis) and −log10(*p* value) (y-axis). The dashed line represents the Bonferroni threshold, and the most significant gene-phenotype associations across all significant tissues are text-labeled.

We investigated the potential functional role of the 27 TWAS genes using data of 1404 clinical phenotypes for *N* = 70,439 individuals of European ancestry using the Vanderbilt Biobank, BioVU (see Methods; Fig. 4 and Supplementary Data 11). Overall, 40 clinical phenotypes were significantly associated with genetically predicted *ABO*, *IFNAR2*, and *CCR1* expression levels; *ABO* accounted for the majority (30 out of 40) of the associations. Top associations with genetically-derived *ABO* gene expression were driven by circulatory system phenotypes, including acute pulmonary heart disease, deep vein thrombosis, other venous embolism and thrombosis, pulmonary heart disease, and acute pulmonary thrombosis and infarction (OR = 1.47; *p* value = 3.97E-11). *IFNAR2* was associated with migraine (OR = 1.35; *p* value = 4.10E-06) and with throat pain (OR = 2.05; *p* value = 2.62E-05; Supplementary Table 2). Across the 17 phenotype categories, we found circulatory system-related phenotypes were enriched for association (see Methods; 7.23-fold enrichment, *p* value = 8.62E-22, Supplementary Data 11). We repeated an enrichment analysis using all association data, regardless of statistical significance, and observed circulatory- and infectious disease-related phenotypes strongly enriched for association signal on average (*p* value <3.9E-44; Supplementary Table 3).

**Fig. 5:**
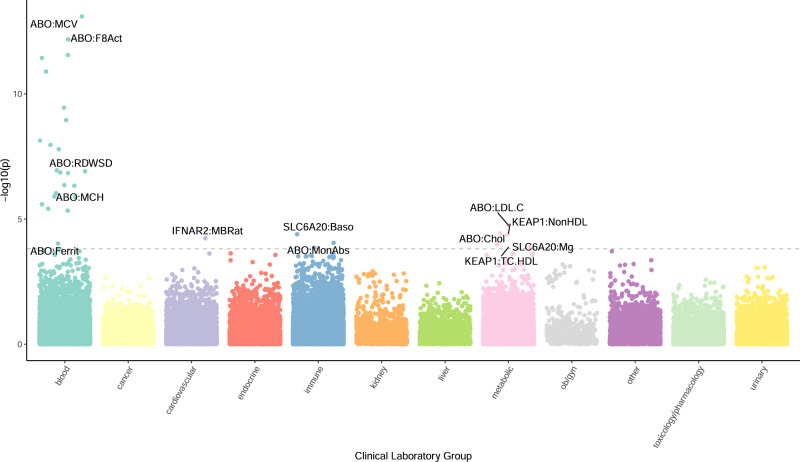
LabWAS Manhattan Plot. Each data point represents laboratory-trait associations with genetically regulated expression of gene-tissue pairs. The data points are grouped and color-coded by clinical laboratory-test groups (x-axis) and −log10(*p* value) (y-axis). The dashed line represents the Bonferroni threshold, and the most significant gene-laboratory trait associations across all significant tissues are text-labeled.

Next, we focused on laboratory results for *N* = 70,337 individuals of European ancestry using the Vanderbilt Biobank, BioVU (see Methods). For the 323 laboratory traits tested, we found 32 labs significantly associated with four genes (*ABO, IFNAR2, KEAP1*, and *SLC6A20*; *p* value <1.55E-04; see Fig. 5 and Supplementary Data 12). Of these, *ABO* captured 27/32 significant associations (mean OR = 1.33; *p* value = 8.02E-14 < 1.40E-04). Genetically-predicted *ABO* expression was associated with various measures of blood and platelet count, coagulation factors, and ferritin in blood, as well as labs measuring immune and metabolic function (Supplementary Data 12). Genetically-predicted *IFNAR2* expression was negatively associated with creatine kinase (OR = 0.89; *p* = 5.85E-05; Supplementary Data 12). Genetically-predicted *KEAP1* expression was positively associated with total cholesterol, and non-high-density lipoprotein levels (beta = 0.50, *p* = 3.72E-05). *SLC6A20* genetically-predicted expression was negatively associated with basophil volume in the blood (beta = −0.19, *p* = 4.04e-5) and magnesium volume in serum/plasma (beta = −0.26, *p* = 1.04e-4). Across the 12 broad lab definitions in our data, among significant LabWAS findings we observed enrichment for blood-related lab measurements (see Methods; 4.21-fold enrichment, *p* value = 1.23E-11; Supplementary Table 4). When extending enrichment analyses to all associations, we found blood- (see Methods; *p* value = 9.23E-22; Supplementary Table 5) and immune-related labs (*p* value = 2.81E-14) displayed the greatest enrichment, with toxicology-, urinary-, and cancer-related labs exhibiting significant depletion of signal (Supplementary Table 5).

### Cross-ancestry phenotypic comparisons

To perform cross-ancestry validation of the clinical and laboratory phenotypes implicated in the European-based results, we performed phenome-wide association study (PheWAS) and LabWAS in the *N* = 15,123 individuals of African ancestry in the BioVU records. Of the 32 identified laboratory measures, we found 22 *ABO-*associated labs replicated at nominal levels (p value <0.05) with five replicating after adjusting for the number of tests performed (*p* value <0.05/32). We attribute a lack of statistical power to the 17 phenotypes that did not replicate after multiple testing corrections. Effect sizes across ancestries were highly concordant (slope = 0.87, 95CI [0.73, 1.01], *p* value = 1.66-13; see Methods; Supplementary Data 12 and Supplementary Fig. 6), with no individual gene/lab pair demonstrating evidence of significant heterogeneity (*p* value <0.05/32). Considering the 40 clinical phenotypes identified in participants of European ancestry, we found none that replicated considering a Bonferroni-corrected significance in African Ancestry individuals. This was largely due to reduced statistical power from smaller sample sizes, as estimated effect sizes were similar across ancestries (slope = 0.33, 95CI [0.25, 0.41], *p* value = 6.72E-10; see Methods**;** Supplementary Data 11 and Supplementary Fig. 6). These cross-ancestry analyses suggest similar effects of predicted expression on relevant clinical and laboratory phenotypes.

We next sought to test cross-ancestry replication of identified phenotypes in the Pan-UK Biobank and Biobank Japan. Briefly, we performed PheWAS using LD-independent eQTL/sQTL SNPs (see Methods) of TWAS-identified genes with significant phenotype associations in GReX-based PheWAS from BioVU. We identified 233 FDR significant SNP-phenotype results dominated by the associations between *ABO* (214/233, 91.8%) SNPs and blood differential tests such as basophils and monocytes. A subset of 80/233 (34.3%) FDR significant associations also were nominally significant in at least one population of non-European ancestry (*p* value <0.05; Supplementary Data 13, 14). There were six instances of FDR significant effect estimate heterogeneity across ancestries, all of which involved *ABO* SNPs and the biomarker alkaline phosphatase or erythrocyte properties. We next tested how significant EUR effect estimates reflect SNP effects across ancestries. We found that EUR SNP effects significantly predicted SNP effects in six global ancestry groups (maximum prediction in AMR; Supplementary Data 15 and Supplementary Fig. 7).

## Discussion

COVID-19 disease is characterized by wide variability in presentations and severity. We integrated multitiered regulatory information with publicly available variant-level data to identify genes associated with COVID-19 related hospitalization. To investigate the potential clinical relevance of these findings, we performed a phenome-wide and lab-wide assessment of the genetically predicted mRNA expression value of each gene that was significantly associated with COVID-19 related hospitalization. We further examined these associations across diverse ancestries and found nominal replication of blood cell traits in diverse ancestral cohorts of Pan-UKBB, BBJ, and an African American population in BioVU.

All three TWAS approaches (mRNA expression, splicing, and protein expression) identified two genes—*ABO* at 9q34.2 and *OAS1* at 12q24.13 (Supplementary Fig. 8) PheWAS results implicated *ABO* in several thrombotic and coagulation-related phenotypes. Thrombotic complications are reported to be both risk factors and sequelae to COVID-19 diagnosis. For example, coagulopathic conditions such as venous thromboembolism^12,13^, deep vein thrombosis^14^, and pulmonary heart disease and embolism^15,16^ constitute of more than 30% prevalent disorders in hospitalized COVID-19 patients. Abnormal blood cell indices are a common denominator shared by severe COVID-19^17^ and thrombotic disorders^18^. These phenotypic observations are further supported by lab-trait associations which showed predicted expression of *ABO* was associated with coagulation factor VIII. This factor is critical for thrombotic homeostasis regulated by its carrier protein—von Willebrand factor^19,20^. Analysis in individuals of both European and African ancestry supported predicted *ABO* expression associating with blood differential tests including mean corpuscle volume, monocyte count, and erythrocytes. *ABO* gene encodes for blood type, and several genes have reported association of blood groups with COVID-19 infection risk^21–26^. *ABO* variants affect the von Willebrand factor and factor VIII levels^27^. Studies have also reported differing prevalence of thrombotic and vascular dysfunction in different blood groups, making the relationship of *ABO* with COVID-19 difficult to ascertain^28–30^. Furthermore, we also observed KEAP1, which is a regulator of cholesterol synthesis^31^. The *ACE2* receptor for SARS-Cov-2 viral binding and uses lipids as docking sites^32^. Increased viral binding affects the instability of the arterial vasculature. Our identification of *KEAP1* through splicing regulation in heart tissue and transcriptomic-phenotype association with cholesterol traits aligns with the hypothesized role of cholesterol in COVID-19 pathogenicity^32^. Furthermore, several studies have reported obesity as a causal risk factor to COVID-19 severity^33–35^.

We observed that associated phenotypes and gene functions converged on cytokine–cytokine receptor signaling involved in inflammatory response (e.g., *CXCR6, CCR9, CCR5, XCR1, IFNAR2, IL10RB*), and on JAK-STAT signaling pathways involved in antiviral host response (e.g., *IFNAR2, OAS1, OAS3*). *IFNAR2* encodes the interferon-alpha/beta receptor beta chain and is responsible for stimulating interferon response which is critical for antiviral immunity previously observed in influenza viral infection^36^. *IFNAR2* is hypothesized to modulate the immune response to COVID-19^37–39^ and interferon deficiency is reportedly associated with severe symptoms of COVID-19^40–42^. These findings are reinforced by results showing that reduced expression (observed in 14 of the 16 significant tissues) of *IFNAR2* is associated with COVID-19 related hospitalization. Given its known function^43^, evidence supports a direct role of *IFNAR2* in an innate antiviral response to COVID-19^37–42^. Genetically predicted expression of *IFNAR2* was associated with migraine in BioVU patients without severe COVID-19. Given that more than 10% of the COVID-19 diagnosed individuals requiring hospitalization reported migraine and headache symptoms^44,45^, reduced host expression of *IFNAR2* may also modulate risk for migraine symptoms in the context of severe COVID-19 infection.

For the pathways identified, we observed a negative TWAS association signal on average for genes in lung tissues, reflecting downregulation for all 13 pathways except the JAK-STAT signaling pathway. The *IL10RB* and *IFNAR2* share an inverse relationship for viral infection, wherein macrophages have been reported to secrete higher IL10 in comparison to IFNAR2^46^. Interestingly, we found cytokine and chemokine genes (*CCR2, CCR3, IFNAR2,* and *OAS1*)^47^ were downregulated^48^ in lung and whole blood, while *IL10RB* and *OAS*3 presented the opposite directions of dysregulation (Supplementary Fig. 4). These inflammatory pathways are further supported by a gene-regulatory network of immunomodulating TFs—*NRF1* and *IRF* that target identified genes (Supplementary Fig. 5). Furthermore, we found evidence for *GNL3* eQTL colocalizing with pulmonary function traits such as FEV and FVC which are decreased in COVID-19 patients^49^. GNL3 is involved in Wnt-B-catenin signaling and affects epithelial-mesenchymal transition, and increased expression is associated with lower survival in lung carcinoma^50^.

In summary, our findings are consistent with a model where genetic variations in immunogenetic loci reported by the previous GWAS^6,8,9^, are associated with hospitalization of COVID-19 via their impact on transcriptomic regulation and its downstream consequences. Furthermore, the transcriptome-wide analysis identified six novel loci, among others, consisting of genes—*IFIT3*, *KEAP1*, and *GNL3* demonstrating their putative association with COVID-19 hospitalization outcome. We further validated previous and novel associations in a multi-ancestry cohort from Vanderbilt BioVU, showing that regulatory variation of identified targets is associated with clinical markers of inflammation, muscle weakness, and pulmonary events. Our cross-ancestry analysis showed how regulatory variants present correlated effect sizes among COVID-19-related phenotypes, expanding our understanding of the interpopulation variability in the susceptibility to the disease.

Previous studies have identified *ACE2* and *TMPRSS2* as targets of SARS-Cov-2 entry and pathogenesis^4^. While we did not observe a significant association between genetically regulated expression of *ACE2* and *TMPRSS2* with COVID-19 hospitalization in our data, this difference can be explained by the different phenotypic definitions. Our study focused on the severity in the host response to SARS-Cov-2 infection instead of the presence/absence of COVID-19 diagnosis. Furthermore, we investigated expression attributed to QTLs, instead of overall gene expression as captured by RNA-sequencing or microarray studies. This may indicate *ACE2* and *TMPRSS2* transcriptomic changes are related to the consequences of SARS-Cov2 and not to the molecular predisposition to develop severe COVID-19 symptoms.

Our results are consistent with previous studies investigating the impact of inflammation on severe COVID-19 outcomes; however, we note there are limitations. First, TWAS analyses rely on SNP-based predictive models of mRNA and alternative splicing trained using mostly European-ancestry individuals in GTEx v8^51–53^. While consistent with the ancestry makeup of COVID-19 HGI GWAS^5^ (https://www.covid19hg.org/), applying these models to non-European individuals (e.g., African Americans in BioVU) will result in loss of power or bias due to different underlying linkage disequilibrium patterns. Second, TWAS uses mRNA, alternative splicing, or protein levels in bulk tissue, with cell-type effects likely to be missed^54^. Third, TWAS assumes additivity of SNP effects on gene expression and downstream hospitalization risk, which ignores the possibility of epistatic and gene-environment interactions contributing to COVID-19 related hospitalization risk. Fourth, while it would be ideal to compare risk and severity prediction using the measured expression, our study leverages large-scale functional datasets to maximize statistical power for TWAS using predicted gene expression. Fifth, our work integrates eQTL from multiple tissues in a joint model to increase statistical power^55–57^. Multiple lines of evidence have demonstrated shared regulatory architectures across tissues;^58–60^ however, we note that tissues unrelated to the pathophysiology of COVID-19 related hospitalization do not necessarily harbor risk, but reflect expression patterns correlated with those found in disease-relevant tissues. Finally, our study focuses on the host genetic factors that contribute to severe COVID-19 but did not incorporate the social determinants of health that are known to influence risk for severe COVID-19. The biological insights identified here should not be interpreted as explanatory factors for the disparity, but instead as key genomic pathways potentially modulating host response to SARS-Cov-2 across populations.

Functional studies of key genes identified are needed to identify mechanisms through which these genes influence COVID-19 related hospitalization. Additionally, while well-powered molecular genetic datasets in diverse populations often lag behind European-ancestry counterparts, massive collaborative science efforts such as the HGI-19 continue to accumulate new datasets to address this discrepancy. Leveraging population eQTL/spQTL/pQTL data with ancestry-matched COVID-19 GWAS will be crucial in identifying and understanding mechanisms underlying COVID-19 related hospitalization. In conclusion, our work raises specific hypotheses relating host genetic variation to the symptom and lab-trait profiles thereby focusing efforts for future drug repurposing and therapeutic discovery research.

## Methods

### COVID19-HGI genome-wide association summary statistics

We downloaded GWAS summary statistics for severe COVID-19 outcomes meta-analyzed across 21 studies (hospitalized *N* = 7885; population *N* = 961,804). A detailed description of the contributing studies, meta-analyses, and primary GWAS results for several COVID-19-related phenotypes are presented at https://www.covid19hg.org/results/, specifically the Freeze 4-October 2020 results File: COVID19_HGI_B2_ALL_leave_23andme_20201020.txt.gz. Summary statistics did not include 23&Me cohort results, and their sample size was removed from the final sample reported. Genome-wide association statistics consisted of inverse-variance meta-analyzed log-odds ratios and their standard errors to compute a final Wald statistic and *p* value. Most of the individuals of the contributing studies to meta-analysis genomic study were of European descent (93%). We performed strict quality control on GWAS data, by filtering statistics at palindromic variants, harmonizing variants with GTEx v8 European-panel genotypes^51^. Our quality control procedure resulted in a final count of 10,340,768 autosomal genetic variants with summary statistics.

### TWAS and spTWAS using models of predicted gene and alternative splicing expression

To perform TWAS and spTWAS we leveraged pretrained prediction models fitted in GTEx v8 data (49 tissues, *N* = 838) using the fine-mapping software DAP-G with a biologically informed prior, Multivariate Adaptive Shrinkage in R (MASHR). For detailed information regarding molecular, genetic, and phenotypic data in GTEx v8, please see ref. ^51^. Prediction models for each tissue were integrated with COVID19-HGI GWAS data using the software S-PrediXcan^56^. In total, we tested 655,563 and 1,728,429 models of total expression and alternative splicing, respectively for association with severe COVID19 outcomes, however, owing to the significant amount of correlation across tissues, we used a per-tissue Bonferroni correction threshold in our multi-tissue analyses (see Supplemental Data 2). To combine association statistics across all tissues while adjusting for tissue–tissue correlation, we used S-MultiXcan^55^. For 22,206 genes, we also performed a joint multi-tissue approach using S-MultiXcan which accounts for tissue correlation and boosts statistical power. Here, we applied a single Bonferroni correction of 0.05/22,206.

### PWAS using models of predicted expression

To perform a protein-wide association study (PWAS) using predicted protein expression, we fitted predictive models using genetic and plasma proteins from European-ancestry individuals in the INTERVAL study (*N* = 3301)^10^. We performed quality control on genotype data and kept only biallelic SNPs with MAF ≧0.01, HWE p > 5e-5, imputation quality INFO >0.6, and were annotated in HapMap3. Plasma proteins had undergone strict quality control and adjustment in the original study^10^. We fit predictive 3222 predictive models for 3170 proteins using genotypes within 1 Mb flanking the gene body (i.e., ±500 kb gene start and stop). For measured proteins consisting of multiple monomers (i.e., dimer, trimer, etc.), we fit multiple predictors, each restricted contributing gene’s region. We included the following covariates into all downstream models of protein abundance: age, sex, duration of blood processing, the top three genotyping PCs, contributing cohort, and top four protein PCs). To reduce the number of tests and increase statistical power, we restricted to genes whose protein levels exhibited evidence of genetic control by testing for nonzero *cis*-heritability (*p* value <0.05) using GCTA. Our final set of local/*cis*-based predictors resulted in 1031 models of protein with significant cis-SNP heritability (*p* value <0.05). We fit penalized linear models using SuSiE^61^ and performed downstream PWAS using the tool FUSION^52^ using quality-controlled COVID19-HGI genome-wide association statistics.

### Allelic specific expression

To determine the allelic effect of GWAS SNPs (*p* value <5e-5) on identified susceptibility genes, we used haplotype-level ASE data with WASP filtering^62^ from the GTEx v8, containing 15,253 samples spanning over 49 human tissues and 838 individuals^51,63^. We used haplotype-aggregated allelic expression generated by phASER^64^. To assess the *cis*-acting regulatory effect of expression imbalance between the alleles in heterozygous individuals, we compared allelic imbalance between the individuals homozygous and heterozygous for each SNP. All individuals with minimum coverage of eight reads (with one pseudocount added) were included. An allelic imbalance was quantified as the log-ratio between the two allelic counts or log allelic fold change (log aFC)^65^ and to ensure robustness to rare variant effects and phasing errors the absolute value of log aFCs are compared, using a one-sided rank-sum test. We used a gene-level Bonferonni correction for the total number of genes tested (*p* value <0.05/21).

### Pathway identification using GSEA and TF-gene network

We tested 27 genes for statistical overrepresentation of pathways using GSEA^11^. We analyzed gene sets of KEGG pathways using ShinyGO^66^. Significance was determined based on a false discovery rate of 5%. To identify the regulatory network, we extracted TF-gene targets using RegNetwork^67^ and created a minimum network, which connects most of the query genes by computing the shortest path between each pair of TF nodes with R packages—igraph and network.

### Colocalization of identified eQTL loci with traits

We investigated novel genes identified in this study and colocalized traits using OpenTarget Genetics^68^, wherein posterior probability (PP) of hypothesis (H4) that two loci are shared under a single causal variant. We report colocalizing traits, only if the phenotype had the keyword “lung” among traits, and H4:PP of >80%. The colocalization was performed using a coloc-R package and described elsewhere^68^.

### Calculating predicted expression in BioVU

To examine the clinical implications of the genes identified in our TWAS analysis in an independent population, we calculated genetically regulated expression for 85,613 individuals in the Vanderbilt biobank, BioVU. The BioVU population consists of individuals who receive care at Vanderbilt University Medical Center and choose to opt-in to the BioVU research study. A detailed description of program operations, ethical considerations, and continuing oversight and patient engagement have been published^69^. Individuals within our BioVU population have an average age of 55.17 years old (median = 59, range = 3 to 112) and 56.9% are female (43.1% male). Genotype information, as well as de-identified electronic medical records, are available for research purposes for these individuals, including information such as International Classification of Diseases, ninth and tenth editions (ICD9/10) billing codes, physician notes, and lab results. Genotype data for the BioVU population was generated using the Illumina MultiEthnic Genotype Array (MEGAEX) for 94,474 individuals. The genotype data were imputed into the HRC reference panel using the Michigan imputation server. Imputed data and the 1000 Genome Project data were combined to carry out principal component analysis (PCA) to identify individuals of European and African ancestry for analysis. We used the best-performance models from PrediXcan, UTMOST, and JTI approaches to impute the expression of 27 genes across 49 tissues^53,70,71^. These models were trained using the GTEx version 8 data^51^.

### BioVU PheWAS

To better understand the phenotypic consequences of dysregulated mRNA expression across our genes of interest, we performed a PheWAS^72^ including patients in the Vanderbilt EHR and linked biobank, BioVU. Phenotypes in BioVU are represented as phecodes, which are assigned as a dichotomous trait and are a hierarchical clustering of the International Classification of Diseases (ICD9/ICD10) codes. For each phenotype, we required a minimum number of 100 cases for inclusion in our PheWAS analyses, which resulted in testing 1404 phecodes in 70,439 individuals of European ancestry and 740 phecodes in 15,174 individuals of African ancestry. We used the PheWAS package in R to perform logistic regression to identify the phecodes that are significantly associated with imputed gene expression after adjusting for sex, age, and the top ten principal components from genetic data to control for population stratification (Denny et al. 2010, 2013). We corrected for the number of tests (i.e., 0.05/1404 = 3.56e-05) to determine statistical significance.

### BioVU biomarker LabWAS

The lab-wide association scan (LabWAS)^73^ allows us to screen clinical lab tests from the Vanderbilt University Medical Center EHR. For each gene identified in the TWAS analyses, we tested the association between its predicted gene expression and all clinical labs. We applied the QualityLab cleaning pipeline^73^ with settings to yield median age-adjusted (residual taken after regressing the cubic splines of age with four knots) inverse normal quantile transformed lab values (to control for skewness and non-normality). We screened across all labs with measurements for at least 100 individuals, which resulted in testing 323 labs in 70,337 individuals of European ancestry and 241 labs in 15,123 individuals of African ancestry. The lab tests are divided into 12 subcategories; blood, metabolic, endocrine, kidney, immune, liver, urinary, OB/gyn, toxicology, cardiovascular, and cancer. Our analyses included the covariates age, sex, and top ten principal components from genetic data to adjust for genetic ancestry. We used a Bonferroni-corrected threshold accounting for the number of labs present in the associations tested (i.e., 0.05/323 = 1.55e-04).

### PheWAS and LabWAS category enrichment analyses

We tested for enrichment of association signals across the clinical categories of phenotypes (laboratories) in PheWAS (LabWAS) in two ways. First, we performed a hypergeometric test using phenotypes/labs that were labeled as significant/not-significant per category. Second, we performed a relaxed test that considers the average magnitude of the association signal in a given category. Here, we computed the mean ${\chi }^{2}$ association statistic per phenotype (laboratory) category and bootstrapped its standard error using 2000 bootstraps. Our enrichment (depletion) statistics for a phenotype (laboratory) category were the difference between its mean ${\chi }^{2}$ from 1 (the expected ${\chi }^{2}$ under the null) divided by the bootstrapped standard error. We used a Bonferroni adjusted *p* value <0.05/*k* to determine enrichment or depletion for either aproach, where *k* = 17 for PheWAS category enrichment tests and *k* = 12 for LabWAS category enrichment tests.

### Cross-ancestry gene effects

We compared effect-sizes of predicted expression on phenotypes and laboratories estimated in European-ancestry patients from the BioVU with estimates obtained from of African-ancestry records. To do so, we performed a weighted linear regression, ${\hat{\beta }}_{A,i}={\hat{\beta }}_{E,i}\alpha +{\epsilon }_{i}$, where ${\hat{\beta }}_{\cdot ,i}$ are the effect-sizes estimated for the ith gene/phenotype or gene/laboratory pair in African- (A) or European-ancestry (E) patients, $\alpha$ is the cross-ancestry relationship, and ${\epsilon }_{i}\sim N\left(0,{\sigma }^{2}\cdot {{{{{{\mathrm{SE}}}}}}}{\left({\hat{\beta }}_{A,i}\right)}^{2}\right)$ is the gene/phenotype-specific (gene/laboratory) noise parameterized by the overall variance ${\sigma }^{2}$ and squared standard-error around the African-ancestry-based estimate. We report estimates of $\hat{\alpha }$ and its 95% confidence intervals assuming normality.

### Cross-ancestry SNP effects

We investigated BioVU phenotypes and laboratory measures significantly associated with TWAS-associated loci for heterogeneous effects in the *trans*-ethnic Pan-UK Biobank (Pan-UKB). The Pan-UKB represents a multi-ancestry analysis of 7221 phenotypes in six continental ancestry groups: African (AFR *N* = 6636), Admixed American (AMR *N* = 980), Centra/South Asian (CSA *N* = 8876), East Asian (EAS *N* = 2709), European (EUR *N* = 420,531), and Middle Eastern (MID *N* = 1599). The Pan-UKB consists of 16,119 GWAS of biological assays, health status, behavioral information, and lifestyle factors. We clumped SNPs in PLINK using eQTL/sQTL *p* values (*q* value ≦0.05) reported in GTEx v8 and limited pair-wise SNP correlations to *r*^2^ = 0.1 over 250 kb windows. Per-tissue clumping resulted in 27 SNPs (*ABO*, *IFNAR2*, *CCR1*, and *SLC6A20*) that were tested with respect to 1571 Pan-UKB phenotypes from the same trait domains detected by PheWAS in BioVU (e.g., circulatory system, digestive disorder, neurological). We used linear models to test for consistency of EUR effect estimates at FDR significant and high confidence SNPs with those estimated in AFR, AMR, CSA, EAS (Pan-UKB and Japan Biobank), and MID populations. Additionally, we used Biobank Japan to verify associations between EUR and EAS-based SNP effects in the Pan-UKB due to increased sample size in the latter. Biobank Japan consists of genetic data for over 200,000 participants and ~120 disease states and quantitative measures (cell type percentage, body mass index, etc.).

### Reporting summary

Further information on research design is available in the Nature Research Reporting Summary linked to this article.

## Supplementary information

Supplementary Information

Description of Additional Supplementary Files

Supplementary Data 1

Supplementary Data 2

Supplementary Data 3

Supplementary Data 4

Supplementary Data 5

Supplementary Data 6

Supplementary Data 7

Supplementary Data 8

Supplementary Data 9

Supplementary Data 10

Supplementary Data 11

Supplementary Data 12

Supplementary Data 13

Supplementary Data 14

Supplementary Data 15

Reporting Summary

## Data Availability

Complete TWAS, spTWAS, PWAS, and allelic imbalance summary statistics results can be accessed here: 10.5281/zenodo.4292567. Covid19 HGI summary statistics: https://www.covid19hg.org/results/r4/. PrediXcan predictive models: https://github.com/hakyimlab/MetaXcan/blob/master/README.md. FUSION PWAS predictive models: https://mancusolab.com/projects/pwas/. Pan-UK Biobank summary statistics: https://pan.ukbb.broadinstitute.org/downloads. Biobank Japan summary statistics: http://jenger.riken.jp/en/result.
