# Supplementary material for: Integrative genomic analyses identify susceptibility genes underlying COVID-19 hospitalization
Source: Nat Commun. 2021 Jul 27;12:4569. doi: 10.1038/s41467-021-24824-z (PMC8316582; doi:10.1038/s41467-021-24824-z)
Supplement: Supplementary file 1 — Supplementary Information [file 41467_2021_24824_MOESM1_ESM.pdf]

Integrative genomic analyses identify susceptibility genes underlying COVID-19 hospitalization

Gita A Pathak[1,2], Kritika Singh*[3,4], Tyne Miller-Fleming [3,4], Frank R Wendt[1,2], Nava Ehsan[5], Kangcheng Hou[6], Ruth Johnson[7], Zeyun Lu[8], Shyamalika Gopalan[8], Loic Yengo[9], Pejman Mohammadi[5,10], Bogdan Pasaniuc[11]‡, Renato Polimanti[1,2]‡, Lea K Davis[3,4]‡, Nicholas Mancuso[8,12]‡***

**Equal contribution*
‡Equal contribution
*** Corresponding author*

Supplemental Note

COVID19 HGI Summary Statistics: We downloaded the publicly available meta-analyzed genome-wide association statistics from the COVID19 HGI (https://www.covid19hg.org/results/), specifically the Freeze 4-October 2020 results File:

COVID19_HGI_B2_ALL_leave_23andme_20201020.txt.gz.

These summary statistics do not include 23&Me cohort results and we acknowledge all the studies comprising of the meta-analysis (https://www.covid19hg.org/publications/).

Supplementary Figures

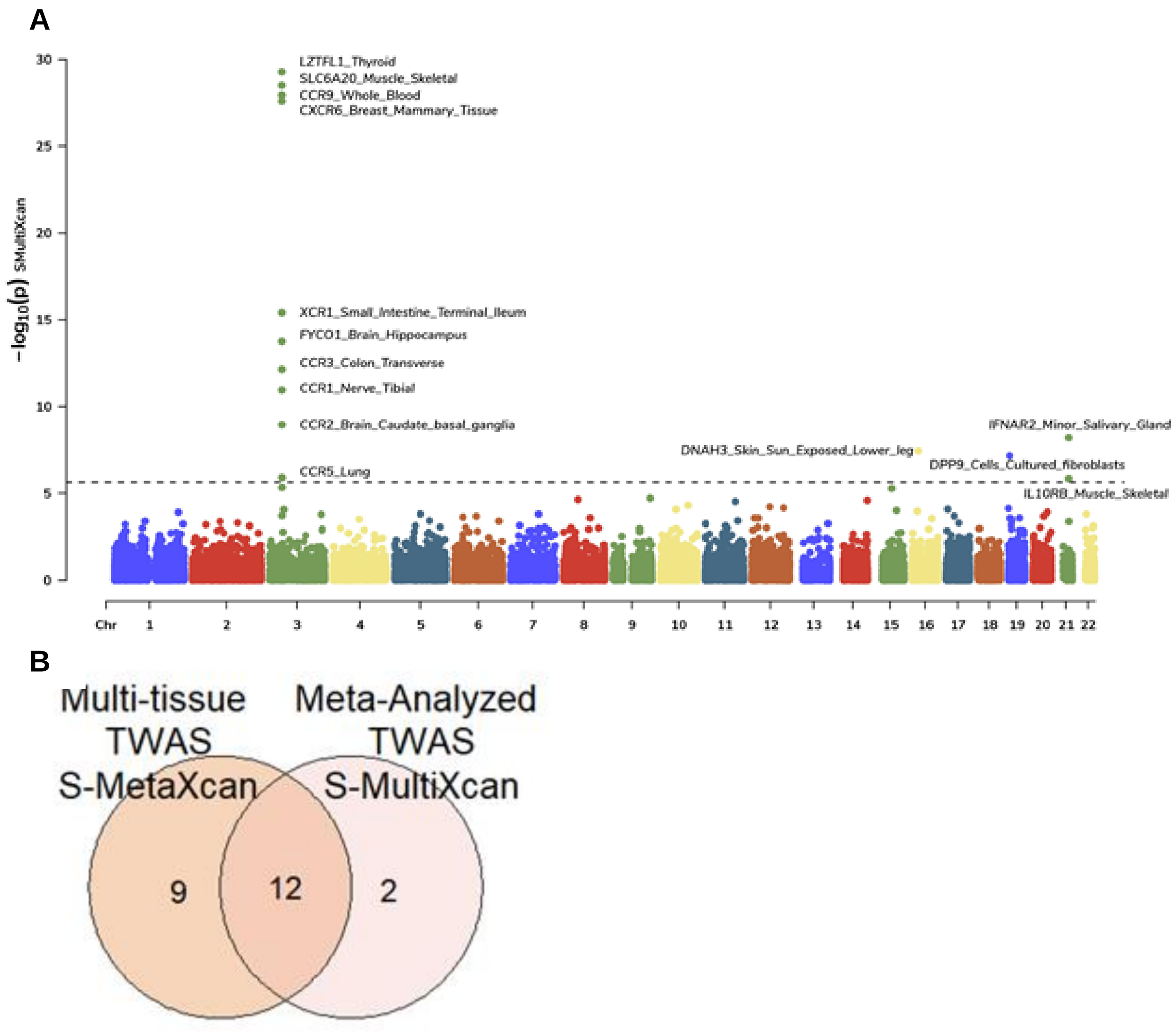

Supplementary Figure 1: Meta-analyzed tissue TWAS of gene expression using SMultiXcan.

A) The Manhattan plot of gene-associations for gene expression transcriptome-wide association study (TWAS). The x-axis is genomic coordinate, grouped by chromosome number, and -log10(p-value) of the association. The dashed line represents Bonferroni threshold, and significant genes and tissues are labelled next to the data point. Full tabular results are available in Supplementary Table 3. **B**) Overlap of significant genes identified from multi-tissue TWAS and meta-analyzed tissue TWAS.

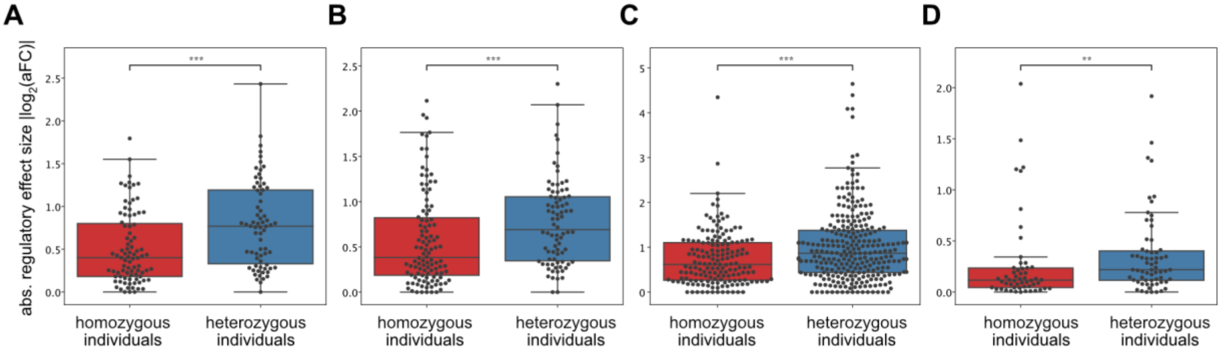

Supplementary Figure 2: Allelic imbalance detected for gene/tissue pairs identified from TWAS, using leading GWAS variants (or closest proxy).

Allelic imbalance of **A**) IL10RB for rs13050728 SNP in brain cerebellar hemisphere (N=157) and **B**) brain cerebellum tissues (N=192), **C**) ABO for rs920065566 in muscle skeletal (N=444) and **D**) OAS3 for rs2269899 in LCL tissue (N=118). Each dot is an individual in GTEX v8 data. The regulatory effect size (y-axis) is the absolute log ratio between the allelic expression of the two haplotypes. Two-sided Mann-Whitney-Wilcoxon p-value annotation legend: null: 5.00E-02 < p <= 1.00; *: 1.00E-02 < p <= 5.00E-02; **: 1.00E-03 < p <= 1.00E-02; ***: 1.00E-04 < p <= 1.00E-03; ****: p <= 1.00E-04. Box represents first quartile, median, and third quartiles. Whiskers represent 1.5 IQR.

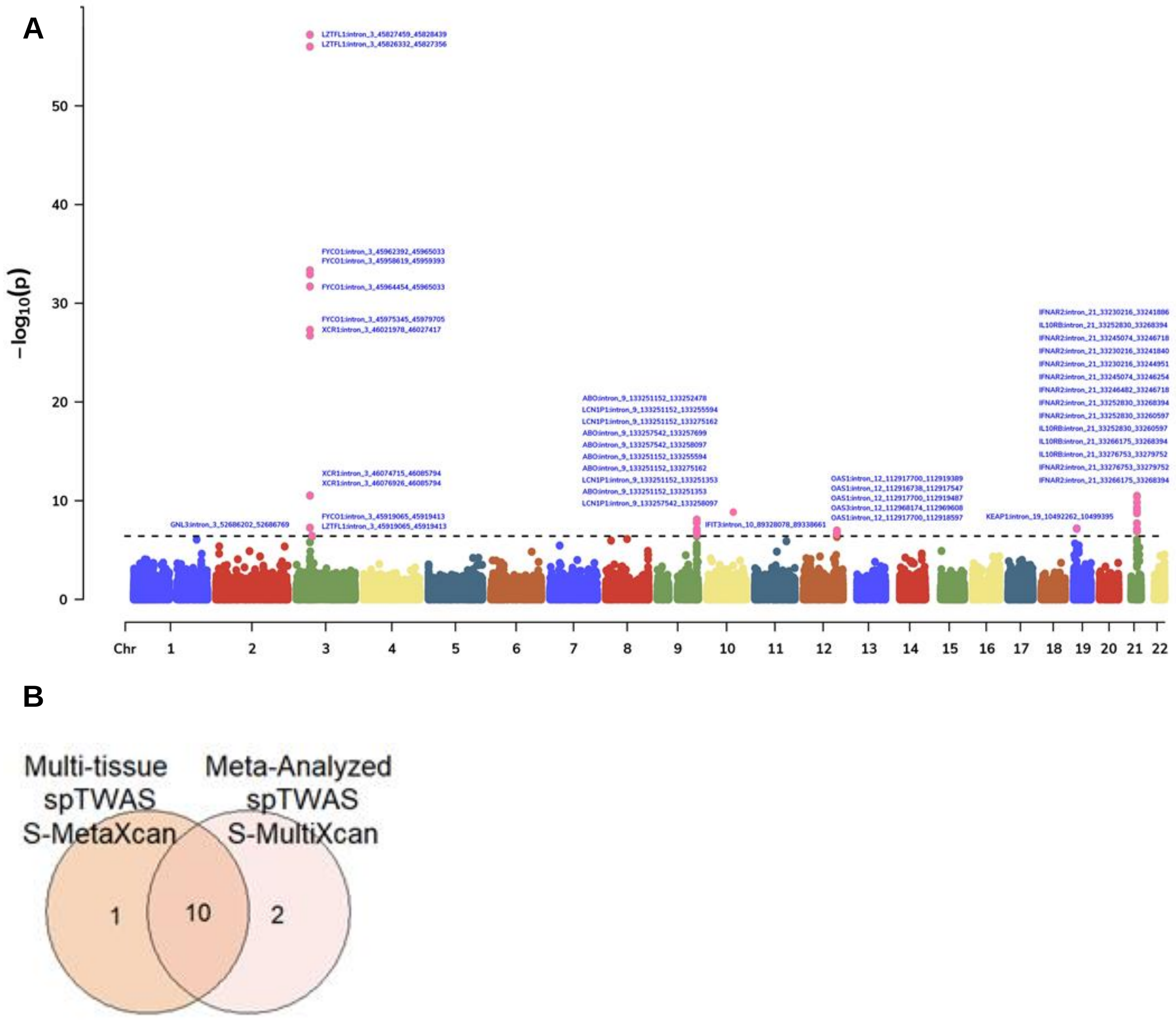

Supplementary Figure 3: Meta-analyzed tissue TWAS of splicing expression using SmultiXcan.

A) Manhattan plot of gene-associations for splicing site expression transcriptome-wide association study (TWAS). The x-axis is genomic coordinate, grouped by chromosome number, and -log10(p-value) of the association. The dashed line represents Bonferroni threshold, and significant splice site position and annotated genes are labelled next to the data point. Full tabular results are available in Supplementary Table 4. **B**) Overlap of significant genes identified from multi-tissue spTWAS and meta-analyzed tissue spTWAS. 10 genes overlap with both analyses, and meta-analysis identified two additional genes – *IFIT3* and *GNL3*. One gene – *LCN1P1* was identified via the multi-tissue approach not observed via meta-analyzed approach.

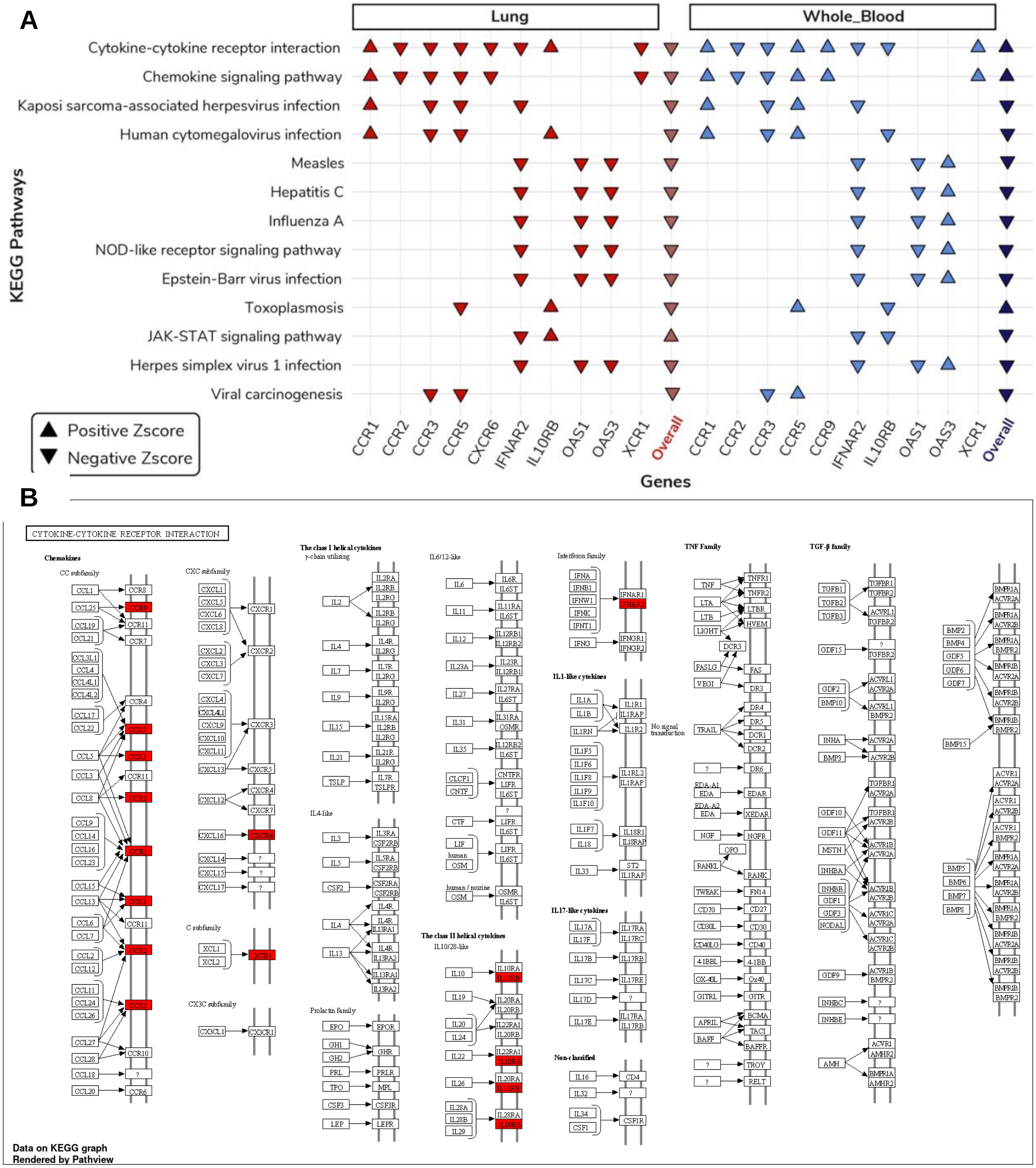

Supplementary Figure 4: KEGG Pathways identified through GSEA.

A) The data points are z-scores of genes (x-axis) for lung and whole blood tissue that belong to KEGG pathways (y-axis; ordered ascendingly based on FDR-p-value). The average z-score across genes for each pathway is shown at the end as ‘Overall’. The p-values are listed in Supplementary Table 12. **B**) Most significant KEGG pathway identified from gene set enrichment analysis (GSEA) is cytokine-cytokine receptor signaling containing nine of the 27 genes identified from TWAS integration.

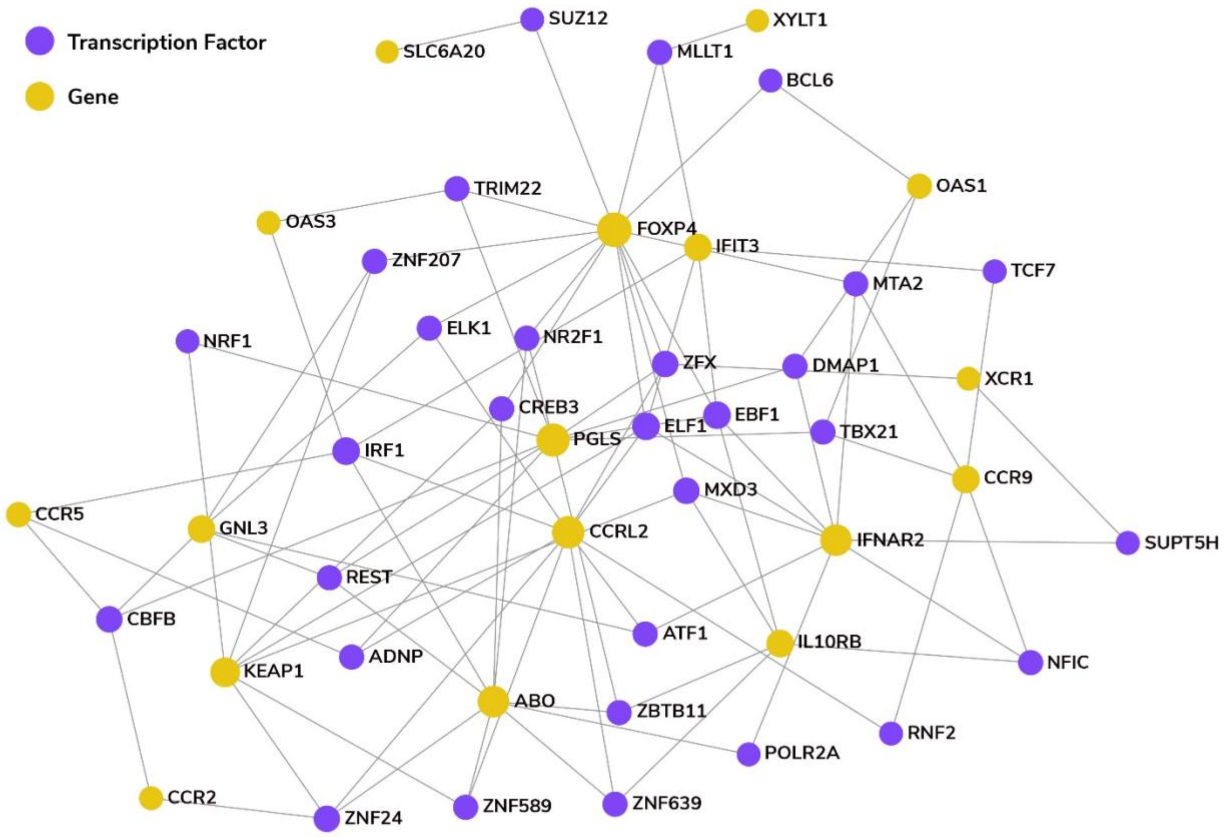

Supplementary Figure 5: Gene regulatory network (GRN) of transcription factors with identified genes.

Transcription-factors (TFs) and gene network identifying TFs that interact with the most number of queried genes.

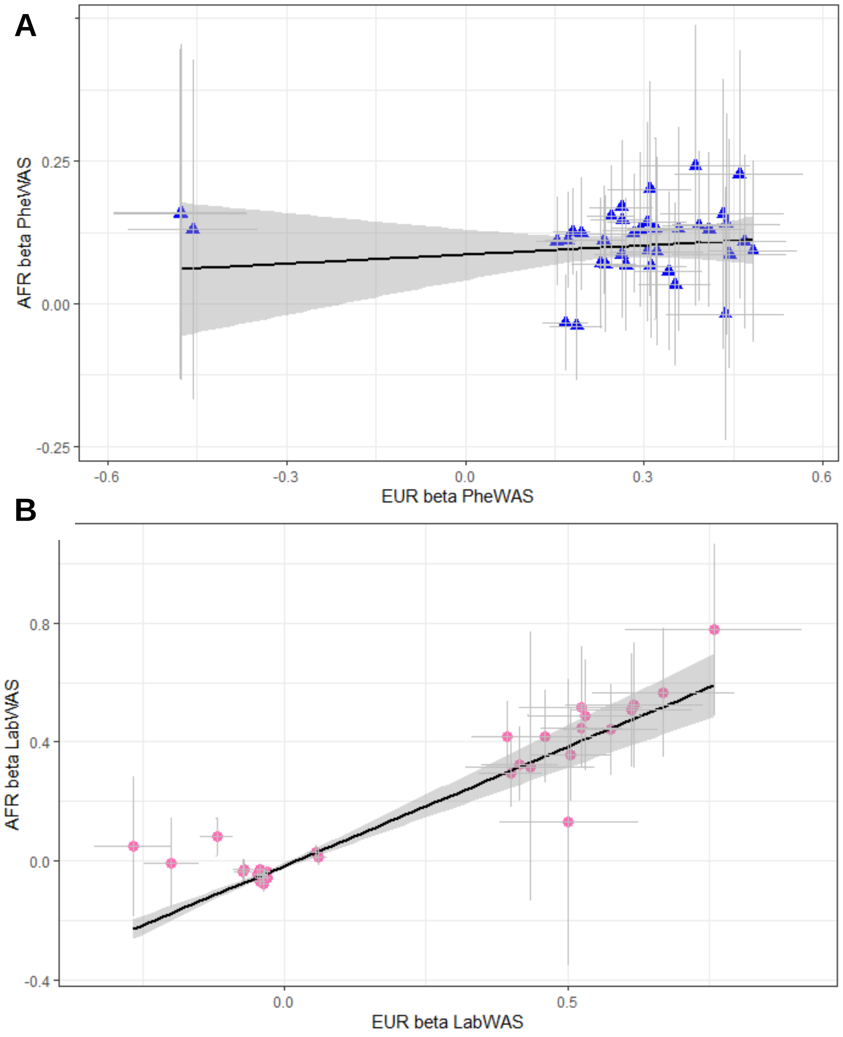

Supplementary Figure 6: Comparing Non-European Effect Sizes in Relation To European Effect Sizes from Lab/PheWAS associations.

Here we compare effect sizes of PheWAS (**A**) and LabWAS (**B**) from BioVU between EUR and AFR ancestry individuals. For the associations that were significant in EUR ancestry, we show cross-ancestry comparisons between the two populations; AFR (y-axis) and EUR (x-axis). The graph shows a linear relationship between beta estimates between populations, highlighting that direction is same, and p-value not surviving multiple testing correction in non-EUR ancestries is due to low sample sizes. Gray bars represent error bars.

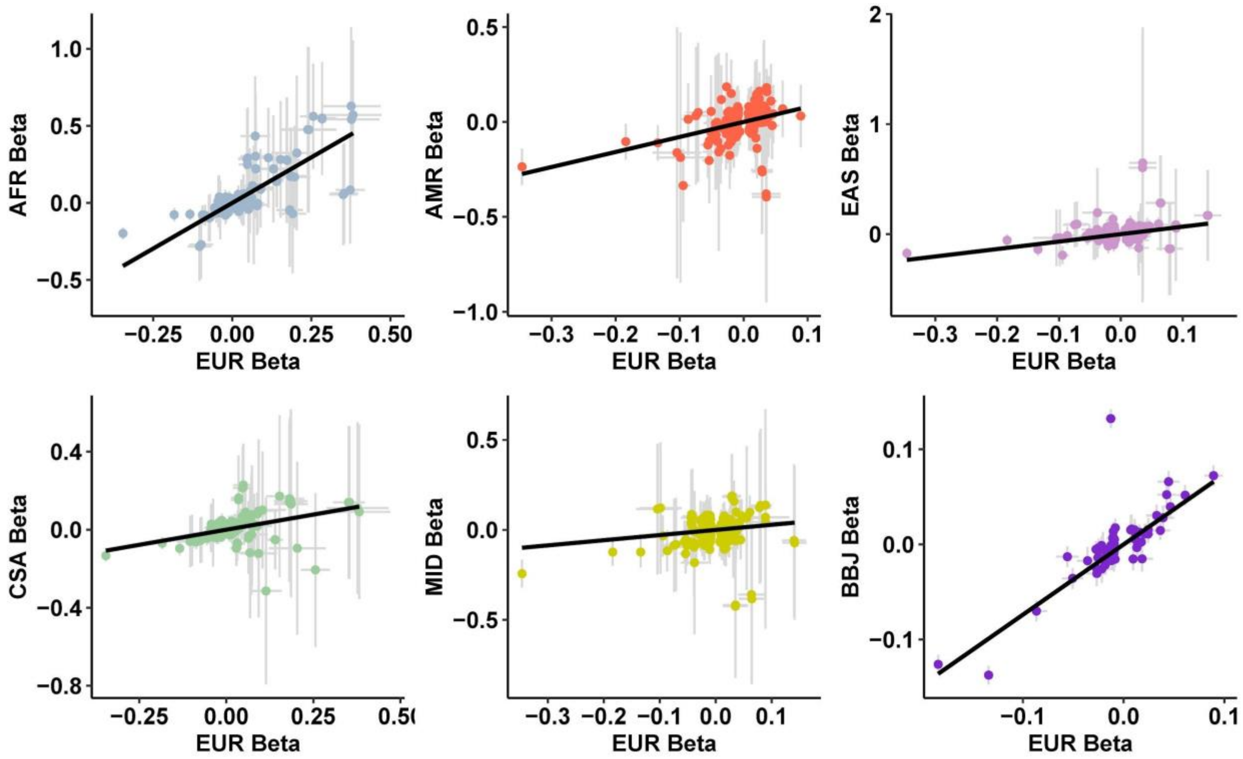

Supplementary Figure 7: Comparing Non-European Effect Sizes in Relation to European Effect Sizes.

Here we compare the effect sizes (beta) of the QTL-SNP-based PheWAS between European (EUR; x-axis) and non-EUR (y-axis; AFR: African, AMR: Admixed American, EAS: East Asian,CSA: Central South Asian,MID: Middle Eastern) ancestry individuals from the Pan-UK biobank. Gray bars represent error bars. The graph shows a linear relationship between beta estimates between populations, highlighting that direction is same, and p-value not surviving multiple testing correction in non-EUR ancestries is due to low sample sizes.

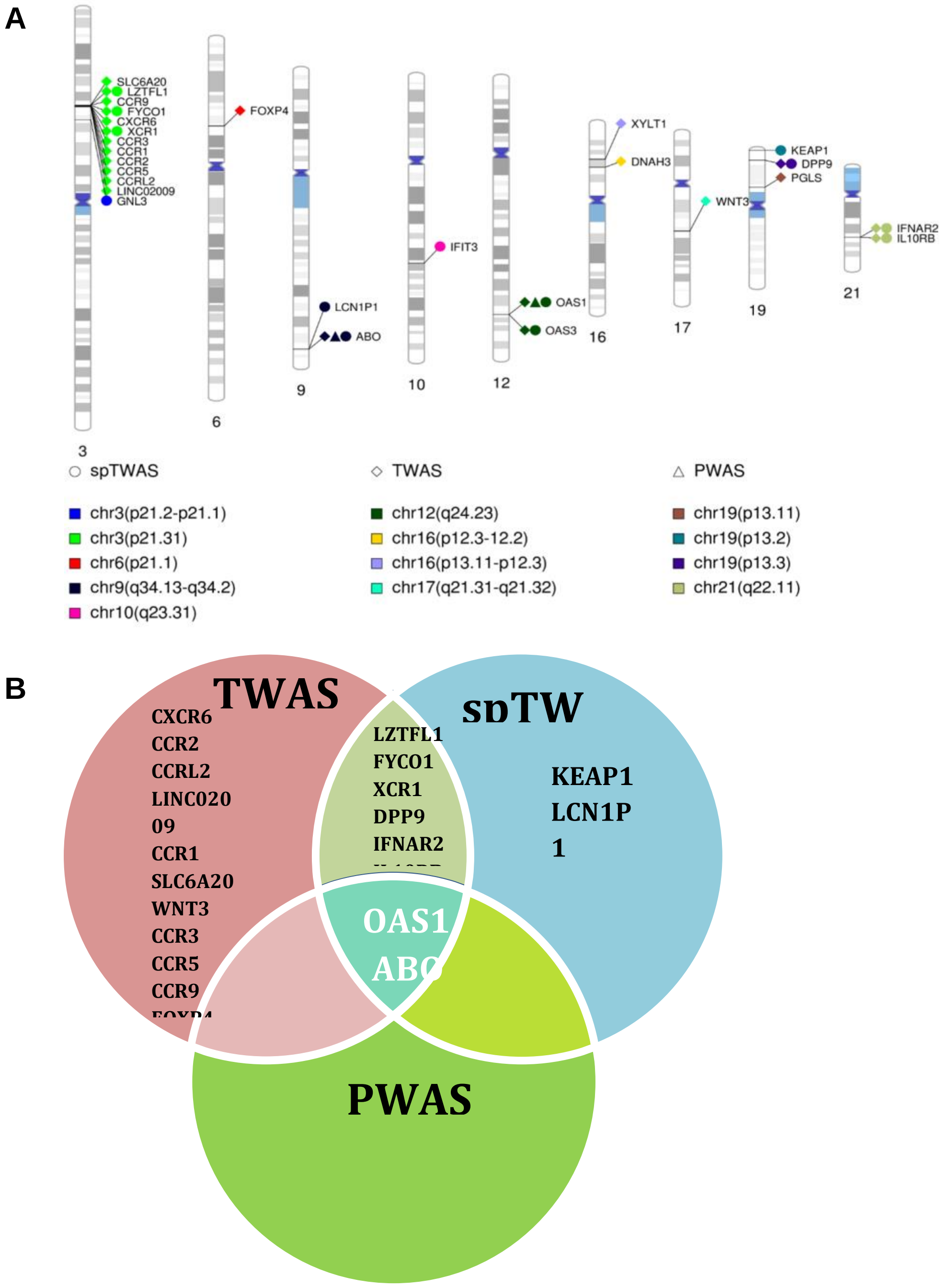

Supplementary Figure 8: Regions identified and overlap of significant genes identified from TWAS, spTWAS and PWAS.

A) The integrative analyses identified 27 genes (labeled) across 13 regions (color coded) shown in the ideogram. **B**) Venn diagram showing overlap of significant genes identified from multi-level TWA studies.

Supplemental Tables

Supplemental Table 1. Gene Set enrichment analysis for Gene Ontology

Enrichment FDR	Functional Category	Genes
3.13E-10	Cytokine-cytokine receptor interaction	CXCR6 CCR9 CCR1 CCR3 CCR5 XCR1 IFNAR2 IL10RB CCR2
9.89E-09	Chemokine signaling pathway	CXCR6 CCR9 CCR1 CCR3 CCR5 XCR1 CCR2
2.63E-04	Kaposi sarcoma-associated herpesvirus infection	CCR1 CCR3 CCR5 IFNAR2
4.05E-04	Human cytomegalovirus infection	CCR1 CCR3 CCR5 IL10RB
1.44E-03	Measles	IFNAR2 OAS1 OAS3
1.68E-03	Hepatitis C	IFNAR2 OAS1 OAS3
1.76E-03	Influenza A	IFNAR2 OAS1 OAS3
1.76E-03	NOD-like receptor signaling pathway	IFNAR2 OAS1 OAS3
2.34E-03	Epstein-Barr virus infection	IFNAR2 OAS1 OAS3
9.96E-03	Toxoplasmosis	CCR5 IL10RB
1.87E-02	JAK-STAT signaling pathway	IFNAR2 IL10RB
2.08E-02	Herpes simplex virus 1 infection	IFNAR2 OAS1 OAS3
2.35E-02	Viral carcinogenesis	CCR3 CCR5

.

Supplementary Table 2. Enrichment of phenotype domains for the significant PheWAS results.

We computed enrichment using two-sided Fisher’s exact test. Fold change reflects odds-ratio.

PheWAS groups	#significant (out of 40)	#total (out of 173551)	p-value	Fold change
circulatory	30	18013	8.62E-22	7.23 fold
neurological	6	9066	1.69E-02	2.87 fold
respiratory	1	9422	3.53E-01	2.17 fold
sense organs	3	11070	4.74E-01	1.18 fold

Supplementary Table 3. Enrichment of phenotype domains for all phenotypes.

Phenotype Group	N tests	Mean TWAS Squared Zscore	SE	Test Statistic	P-value
circulatory system	31017	1.14	0.01	14.00	1.66E-44
infectious diseases	9917	1.24	0.02	13.93	3.90E-44
sense organs	19623	1.15	0.01	12.67	8.33E-37
endocrine/metabolic	26375	1.10	0.01	10.77	4.86E-27
dermatologic	15192	1.14	0.01	10.23	1.52E-24
respiratory	16458	1.12	0.01	9.77	1.44E-22
pregnancy complications	6541	1.18	0.02	9.47	2.84E-21
symptoms	7385	1.16	0.02	9.11	7.89E-20
hematopoietic	9284	1.12	0.02	8.09	6.04E-16
mental disorders	14348	1.07	0.01	5.10	3.39E-07
digestive	27852	1.04	0.01	4.29	1.79E-05
neurological	14981	1.04	0.01	3.05	2.27E-03
injuries & poisonings	16669	0.98	0.01	-2.30	2.15E-02
musculoskeletal	20045	1.01	0.01	1.49	1.37E-01
neoplasms	25531	0.99	0.01	-1.30	1.94E-01
genitourinary	27219	1.00	0.01	-0.34	7.35E-01
congenital anomalies	7807	1.00	0.01	-0.25	8.06E-01

Legend:
Phenotype group: phenotype group tested for association with COVID-19 identified genes
N tests: the number of association tests performed per category
Mean TWAS Squared Zscore: Average TWAS squared z-score within category
SE: Bootstrapped standard error across 2000 re-sampled datasets
Test statistic: (Mean Squared Zscore - 1) / SE; Here the null z-squared is 1.
P-value: Two-sided test under standard Normal

Supplementary Table 4. Enrichment of lab domains for the significant lab-phenotypes.
We computed enrichment using two-sided Fisher's exact test. Fold change reflects odds-ratio.

LabWAS groups	#significant (out of 32)	#total (out of 53055)	P-value	Fold change
blood	23	9064	1.23E-11	4.21 fold
metabolic	5	11277	0.30	1.36 fold
immune	3	11956	0.05	2.4 fold
cardiovascular	1	3994	0.29	2.41 fold

Supplementary Table 5. Enrichment of TWAS association in lab domains for lab-related phenotypes.

LabWAS Group	N tests	Mean TWAS Squared Zscore	SE	Test Statistic	P value
toxicology/pharmacology	3690	0.78	0.02	-12.41	2.37E-35
blood	11685	1.19	0.02	10.03	1.09E-23
urinary	4715	0.83	0.02	-9.59	9.23E-22
immune	15170	1.10	0.01	7.61	2.81E-14
cancer	2050	0.84	0.03	-6.08	1.24E-09
ob/gyn	410	1.53	0.11	4.64	3.45E-06
other	2870	1.05	0.03	1.92	5.49E-02
liver	1230	1.05	0.04	1.46	1.43E-01
endocrine	4305	1.03	0.02	1.46	1.45E-01
metabolic	13940	0.99	0.01	-1.27	2.03E-01
kidney	1230	1.05	0.05	1.13	2.57E-01
cardiovascular	4920	1.00	0.02	0.18	8.58E-01

Legend:
LabWAS group: phenotype group tested for association with COVID-19 identified genes
N tests: the number of association tests performed per category
Mean TWAS Squared Zscore: Average TWAS squared z-score within category
SE: Bootstrapped standard error across 2000 re-sampled datasets
Test statistic: (Mean Squared Zscore - 1) / SE; Here the null z-squared is 1.
P-value: Two-sided test under standard Normal
