# Supplementary material for: Integrative genomic analyses identify susceptibility genes underlying COVID-19 hospitalization
Source: Nat Commun. 2021 Jul 27;12:4569. doi: 10.1038/s41467-021-24824-z (PMC8316582; doi:10.1038/s41467-021-24824-z)
Supplement: Supplementary file 2 — Description of Additional Supplementary Files [file 41467_2021_24824_MOESM2_ESM.pdf]

Description of Additional Supplementary Files

Supplementary Data 1: Significant results for the multi-tissue TWAS using S-MetaXcan.

Supplementary Data 2: Number of TWAS tests per tissue using GTEx v8 data.

Supplementary Data 3: Results of expression TWAS using meta-analyzed across tissue approach using S-MultiXcan.

Supplementary Data 4: Allelic imbalance results for genes using leading GWAS variants (or closest proxy) within 1Mb region.

Supplementary Data 5: Significant results for the spTWAS from multi-tissue approach using S-MetaXcan.

Supplementary Data 6: Number of spTWAS tests per tissue using GTEx v8 data.

Supplementary Data 7: Results of splicing TWAS using meta-analyzed across tissue approach using S-MultiXcan.

Supplementary Data 8: Comparing effect size estimates of genes that were significant using both eTWAS and spTWAS.

Supplementary Data 9: All results for PWAS using FUSION.

Supplementary Data 10: Colocalizing traits using OpenTarget Genetics.

Supplementary Data 11: Significant results for PheWAS using the genetically-regulated gene expression.

Supplementary Data 12: Significant results for LabWAS using the genetically-regulated gene expression.

Supplementary Data 13: PheWAS of eQTL SNPs for the gene/tissues identified from GTEx-based Phe/LabWAS in the Pan-UK biobank.

Supplementary data 14: PheWAS of eQTL SNPs for the gene/tissues identified from GTEx-based Phe/LabWAS in the Biobank Japan and its comparison with Pan-UK biobank.

Supplementary Data 15: Results from Linear Models of Non-European Effect Sizes in Relation To European Effect Sizes.
