# Supplementary material for: Integrative genomic analyses identify susceptibility genes underlying COVID-19 hospitalization
Source: Nat Commun. 2021 Jul 27;12:4569. doi: 10.1038/s41467-021-24824-z (PMC8316582; doi:10.1038/s41467-021-24824-z)
Supplement: Supplementary file 18 — Reporting Summary [file 41467_2021_24824_MOESM18_ESM.pdf]

nature portfolio

Corresponding author(s): Nicholas Mancuso; nicholas.mancuso@med.usc.edu

Last updated by author(s): Jun 18, 2021

Reporting Summary

Nature Portfolio wishes to improve the reproducibility of the work that we publish. This form provides structure for consistency and transparency in reporting. For further information on Nature Portfolio policies, see our Editorial Policies and the Editorial Policy Checklist.

Statistics

For all statistical analyses, confirm that the following items are present in the figure legend, table legend, main text, or Methods section.

n/a	Confirmed	
☐	☒	The exact sample size (*n*) for each experimental group/condition, given as a discrete number and unit of measurement
☒	☐	A statement on whether measurements were taken from distinct samples or whether the same sample was measured repeatedly
☐	☒	The statistical test(s) used AND whether they are one- or two-sided<br>*Only common tests should be described solely by name; describe more complex techniques in the Methods section.*
☐	☒	A description of all covariates tested
☐	☒	A description of any assumptions or corrections, such as tests of normality and adjustment for multiple comparisons
☐	☒	A full description of the statistical parameters including central tendency (e.g. means) or other basic estimates (e.g. regression coefficient) AND variation (e.g. standard deviation) or associated estimates of uncertainty (e.g. confidence intervals)
☐	☒	For null hypothesis testing, the test statistic (e.g. *F*, *t*, *r*) with confidence intervals, effect sizes, degrees of freedom and *P* value noted<br>*Give P values as exact values whenever suitable.*
☒	☐	For Bayesian analysis, information on the choice of priors and Markov chain Monte Carlo settings
☒	☐	For hierarchical and complex designs, identification of the appropriate level for tests and full reporting of outcomes
☒	☐	Estimates of effect sizes (e.g. Cohen's *d*, Pearson's *r*), indicating how they were calculated

Our web collection on statistics for biologists contains articles on many of the points above.

Software and code

Policy information about availability of computer code

Data collection	No custom software was used to collect data for this study.
Data analysis	We performed summary-based TWAS analyses using S-PrediXcan/MetaXcan v 0.6.5 (https://github.com/hakyimlab/MetaXcan) and FUSION (https://github.com/gusevlab/fusion_twas/; commit 6fedd22b47f9dab6a790c7779467f4d40ae57704). We performed LabWAS and PheWAS using the PheWAS R package (https://github.com/PheWAS/PheWAS; version 0.99.5-4). All analyses that summarized results were performed using custom R scripts (version 3.6).

For manuscripts utilizing custom algorithms or software that are central to the research but not yet described in published literature, software must be made available to editors and reviewers. We strongly encourage code deposition in a community repository (e.g. GitHub). See the Nature Portfolio guidelines for submitting code & software for further information.

Data

Policy information about availability of data

All manuscripts must include a data availability statement. This statement should provide the following information, where applicable:

- Accession codes, unique identifiers, or web links for publicly available datasets
- A description of any restrictions on data availability
- For clinical datasets or third party data, please ensure that the statement adheres to our policy

We have a data availability statement reflecting public datasets used in this study.

Field-specific reporting

Please select the one below that is the best fit for your research. If you are not sure, read the appropriate sections before making your selection.

- [x] Life sciences
- [ ] Behavioural & social sciences
- [ ] Ecological, evolutionary & environmental sciences

For a reference copy of the document with all sections, see nature.com/documents/nr-reporting-summary-flat.pdf

Life sciences study design

All studies must disclose on these points even when the disclosure is negative.

Sample size	All analyses performed relied on existing data and as such no a-priori calculations were performed to estimate sample size for a pre-determined power. COVID19 HGI Freeze 4, excluding 23&Me GWAS sample size (7,885 cases and 961,804) was provided by COVID19 HGI consortium. Ancestry-specific sample sizes in PanUK Biobank were provided by PanUKBB team. JBiobank Japan sample sizes were provided by the Biobank Japan consortium. BioVU participate sample sizes (N=85,613; N_EUR = 70,439; N_AFR = 15,174) were determined by simple tabulation.
Data exclusions	No data were excluded from analysis.
Replication	European-ancestry based LabWAS and PheWAS results were repeated in African-ancestry BioVU participants. Of the 32 associated labs identified in European ancestry individuals, 5 replicated at the Bonferroni-adjusted level in African ancestry individuals. We attribute the replication rate to reduced power as effect size estimates were consistent across ancestry groups, after accounting for sample-size/standard errors (see Methods).
Randomization	Samples were not randomized. LabWAS and PheWAS models were adjusted using age, sex, and the top 10 genotype principal components.
Blinding	Blinding is not relevant as all analyses used existing data collected and summarized through COVID19 HGI, PanUKBB, and Vanderbilt BioVU.

Reporting for specific materials, systems and methods

We require information from authors about some types of materials, experimental systems and methods used in many studies. Here, indicate whether each material, system or method listed is relevant to your study. If you are not sure if a list item applies to your research, read the appropriate section before selecting a response.

Materials & experimental systems

n/a	Involved in the study	
x		Antibodies
x		Eukaryotic cell lines
x		Palaeontology and archaeology
x		Animals and other organisms
x		Human research participants
x		Clinical data
x		Dual use research of concern

Methods

n/a	Involved in the study	
x		ChIP-seq
x		Flow cytometry
x		MRI-based neuroimaging
